# *Zaire ebolavirus* surveillance near the Bikoro region of the Democratic Republic of the Congo during the 2018 outbreak reveals presence of seropositive bats

**DOI:** 10.1371/journal.pntd.0010504

**Published:** 2022-06-22

**Authors:** Stephanie N. Seifert, Robert J. Fischer, Eeva Kuisma, Cynthia Badzi Nkoua, Gerard Bounga, Marc-Joël Akongo, Jonathan E. Schulz, Beatriz Escudero-Pérez, Beal-Junior Akoundzie, Vishnou Reize Bani Ampiri, Ankara Dieudonne, Ghislain Dzeret Indolo, Serge D. Kaba, Igor Louzolo, Lucette Nathalie Macosso, Yanne Mavoungou, Valchy Bel-bebi Miegakanda, Rock Aimé Nina, Kevin Tolovou Samabide, Alain I. Ondzie, Francine Ntoumi, César Muñoz-Fontela, Jean-Vivien Mombouli, Sarah H. Olson, Chris Walzer, Fabien Roch Niama, Vincent J. Munster

**Affiliations:** 1 Paul G. Allen School for Global Health, College of Veterinary Medicine, Washington State University, Pullman, Washington, United States; 2 Virus Ecology Section, Laboratory of Virology, Rocky Mountain Laboratories, National Institutes of Allergy and Infectious Diseases, National Institutes of Health, Hamilton, Montana, United States; 3 Wildlife Health Program, Wildlife Conservation Society, Brazzaville, Republic of the Congo; 4 Département de la Recherche et de la Production, Laboratoire National de Santé Publique, Brazzaville, Republic of the Congo; 5 Bernhard Nocht Institute for Tropical Medicine, Hamburg, Germany; 6 German Centre for Infection Research (DZIF), Partner Site Hamburg-Luebeck-Borstel, Germany; 7 Direction de la Santé Animale, Ministére de L’Agriculture et de L’Élevage, Brazzaville, Republic of the Congo; 8 Faculty of Sciences and Techniques, Université Marien N’Gouabi, Brazzaville, Republic of the Congo; 9 Fondation Congolaise pour la Recherche Médicale, Brazzaville, Republic of the Congo; 10 Institute of Tropical Medicine, University of Tübingen, Tübingen, Germany; 11 Health Program, Wildlife Conservation Society, New York, New York, United States; 12 Research Institute of Wildlife Ecology, University of Veterinary Medicine, Vienna, Austria; Saudi Ministry of Health, SAUDI ARABIA

## Abstract

On the 8^th^ of May, 2018, an outbreak of Ebola virus disease (EVD) was declared, originating in the Bikoro region of the Democratic Republic of the Congo (DRC) near the border with neighboring Republic of the Congo (ROC). Frequent trade and migration occur between DRC and ROC-based communities residing along the Congo River. In June 2018, a field team was deployed to determine whether *Zaire ebolavirus* (Ebola virus (EBOV)) was contemporaneously circulating in local bats at the human-animal interface in ROC near the Bikoro EVD outbreak. Samples were collected from bats in the Cuvette and Likouala departments, ROC, bordering the Équateur Province in DRC where the Bikoro EVD outbreak was first detected. EBOV genomic material was not detected in bat-derived samples by targeted quantitative reverse transcription-polymerase chain reaction or by family-level consensus polymerase chain reaction; however, serological data suggests recent exposure to EBOV in bats in the region. We collected serum from 144 bats in the Cuvette department with 6.9% seropositivity against the EBOV glycoprotein and 14.3% seropositivity for serum collected from 27 fruit bats and one Molossinae in the Likouala department. We conclude that proactive investment in longitudinal sampling for filoviruses at the human-animal interface, coupled with ecological investigations are needed to identify EBOV wildlife reservoirs.

## Author summary

*Zaire ebolavirus* (Ebola virus, (EBOV)), first characterized more than 40 years ago, is a causative agent of Ebola virus disease (EVD) which is associated with viral hemorrhagic fever. EBOV is thought to be transmitted to humans through contact with wildlife. Though bats are suspected to be the hosts that maintain EBOV in nature, it is currently not known which bat species serve as the reservoir. Identifying the wildlife species that maintain EBOV in nature would facilitate the development of strategies to mitigate the risk of EBOV transmission to humans from wildlife. In this manuscript, we describe the results of surveillance efforts to detect Ebola virus in wildlife near a contemporaneous outbreak of EBOV disease in humans. We detected evidence of recent EBOV infection in bats near the location of the EVD outbreak through serological techniques, but we did not find evidence of active EBOV infection in bats. These data support the need for more proactive surveillance in wildlife in EBOV endemic regions.

## Introduction

More than forty years have passed since the characterization of *Zaire ebolavirus* (Ebola virus (EBOV)) as a causative agent of Ebola virus disease (EVD), and still the wildlife reservoir remains elusive. Since that time there have been dozens of EVD outbreaks caused by multiple viruses in the *Ebolavirus genus* including Ebola virus, Sudan virus, and Bundibugyo virus, with EVD outbreaks primarily originating in Central Africa. Bats have been implicated as the most likely wildlife reservoir of EBOV [1] and other related filoviruses [2,3,4,5,6,7]. Despite many efforts to identify the reservoir of EBOV, only a single group has detected and published on EBOV nucleic acids in wild bats [1]. Following extensive sampling in a region where several EBOV spillover events had been detected in humans and in great apes from 2001–2003 at the border between northern Gabon and Republic of Congo (ROC), Leroy *et al*. (2005) detected Ebola virus nucleic acids in 13 bats belonging to three species of fruit bat, *Hypsignathus monstrosus*, *Epomops franqueti*, and *Myonycteris torquata* [1]. Since that time there have been no new EVD outbreaks in Gabon or ROC and no published data has replicated the findings of Leroy *et al*. 2005[1]. Identifying the reservoir or reservoirs of EBOV in the Congo basin continues to elude researchers, hindering the effort to identify the ecological and socio-economic factors that contribute to spillover events.

Outbreaks of EVD originating in Équateur Province, Democratic Republic of the Congo (DRC) have been reported three times in recent years with an EVD outbreak Jul—Nov, 2014, resulting in 66 EVD cases and 49 deaths [8], May—Jul, 2018, in Bikoro, DRC, resulting in 54 EVD cases and 33 deaths [9,10], and May–Nov, 2020 with 130 cases and 55 deaths reported [11]. The Équateur Province in northwestern DRC is adjacent to the Congo River which delineates the border between DRC and neighboring Republic of the Congo (ROC); there is frequent trade and migration between the countries along the Congo River.

**Fig 1:**
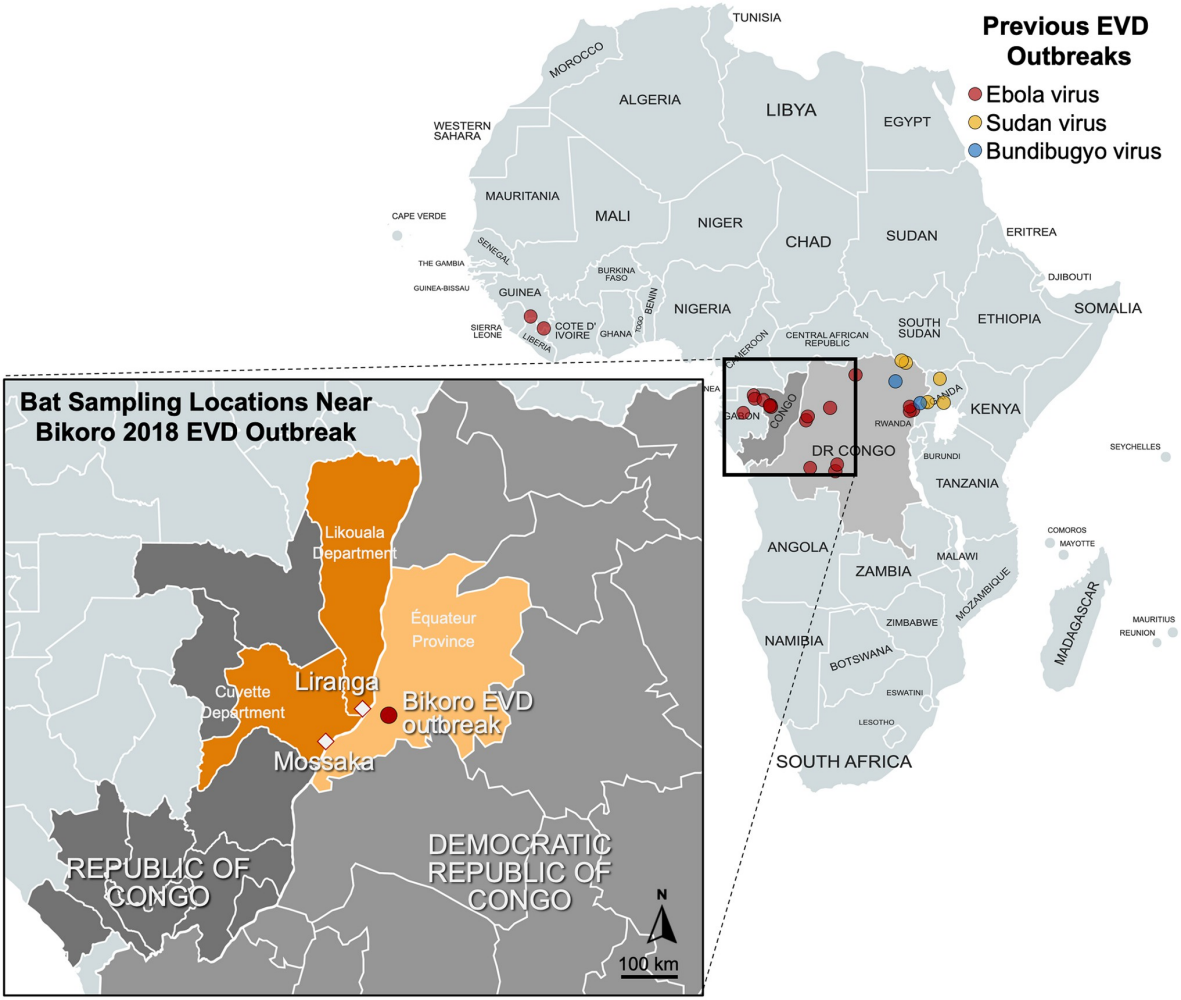
Locations of historical Ebola virus disease outbreaks in Africa associated with *Zaire ebolavirus* (Ebola virus, red), Sudan virus (yellow), or Bundibugyo virus (blue). Inset map shows the sampling locations in Mossaka and Liranga, Republic of Congo, relative to the 2018 Ebola virus disease outbreak in Bikoro, Democratic Republic of Congo. A total of 31 bats were sampled at the Liranga field sites and 165 bats were sampled at the Mossaka field site. Base layer map data sourced through Natural Earth http://www.naturalearthdata.com/.

Following the declaration of an outbreak of EVD on the 8^th^ of May, 2018, in the market town Bikoro, Équateur Province, DRC, a field team was deployed to the municipalities, Mossaka and Liranga, ROC, near the Bikoro EVD outbreak in DRC to conduct surveillance in bats for EBOV (Fig 1). We prioritized sampling from synanthropic bat species including bats in the subfamily Molossinae, *Mops condylurus* (Angolan free-tailed bat) and *Chaerephon pumilus* (little free-tailed bat), which are commonly associated with human dwellings in central Africa. *M*. *condylurus* bats have been implicated as a plausible source of the 2013 EBOV spillover in Guinea [12], while genomic material for a related Ebolavirus, Bombali virus, has been recovered from both *M*. *condylurus* and *C*. *pumilus* bats [4, 13, 14].

## Methods

### Ethics statement

All field work was performed under the approval of L’Institut National de Recherche Forestiére, Brazzaville, ROC (Permit No. 260) and under approval of the Rocky Mountain Laboratories, Division of Intramural Research, National Institute of Allergy and Infectious Diseases, National Institutes of Health, Institutional Animal Care and Use Committee (Animal Study Protocol #2018-15-F) and Institutional Biosafety Committee (IBC).

### Sampling

The field team arrived in Mossaka, ROC, and began sampling bats on the 20^th^ of June, 2018, with researchers from the National Public Health Laboratory, Congo-Brazzaville, the Wildlife Health Program, Wildlife Conservation Society, Congo-Brazzaville, and the National Institutes of Health, United States.

Mist nets were deployed in Mossaka, ROC, near the local hospital (Lat: -1.224809, Long: 16.797453) where the Likouala tributary branches from the Congo River known for fish production. Mossaka was selected as a sampling location due to the proximity to the Bikoro EVD outbreak, observed abundance of bats roosting in human structures, large population relative to surrounding municipalities, and the importance of the town as a port for trade on the floating marketplace between ROC and DRC. The town is structured with residential blocks bordering the river with narrow, sandy paths frequented by pedestrians and motorcyclists. We noted the presence of several free-roaming cats, dogs, chickens, goats, and two pigs. Bats were actively hunting mosquitoes and other insects aggregating near artificial light sources throughout the town of Mossaka. In order to minimize both the biohazard risk and potential disruption to the town, we set the mist nets near sources of artificial light in the central courtyard of the Mossaka Hospital after the gates closed at dusk. As the Mossaka Hospital was the largest source of artificial light in the town after 22:00, insectivorous bat hunting activity was high in this clearing. Mist nets were set until 10 bats were captured, then closed until 8 of those bats were sampled and released. We aimed to process approximately 25 bats per sampling night at this location. Mist nets were also deployed in the fishing village, Liranga, ROC, (Lat: -0.655914, Long: 17.606579) which is adjacent to Bikoro, DRC, the location of the EVD outbreak (Fig 1). Liranga was selected as a sampling location as it is geographically situated near the epicenter of the Bikoro EVD outbreak and the open clearings of Liranga are surrounded by disturbed forest which provided an opportunity to sample from fruit bats. The mist nets were set up in a clearing near the riverbank. There were few free-roaming livestock and companion animals in the village, including pigs, goats, chickens, dogs, and cats, as well as an outdoor market with primarily fish. We did not see wild game at the time of sampling in the village. In Liranga, the mist nets were set up at 19:00 and checked every 20 minutes for bats for two nights with 15 bats captured one night and 16 bats captured the second night.

Oral/nasal, urogenital, and rectal swabs were collected into 1mL of viral transport media for each bat sampled, swab samples were then stored the same night in portable -80°C freezers or liquid nitrogen dry shippers before long-term storage at -45°C. Swabs were not collected from 11 bats which were lethally sampled in Liranga from which tissue samples were collected. Blood was collected from 144 bats at the Mossaka field site and 28 bats at the Liranga field site. Blood or blood in pH neutral phosphate buffered saline (PBS), for smaller bats, was aliquoted for molecular surveillance and frozen in -80°C freezers or liquid nitrogen dry shippers the same night before long-term storage at -45°C. Nonlethal blood volume collected from Molossinae bats varied from 5–50 uL depending on size of bat (juvenile Molossinae bats weighed as little as 7 g) and the blood flow which can be influenced by several factors including hydration and temperature. Blood volume collected for each individual was recorded for and transferred from the capillary tube into 500 uL of PBS with 140 uL immediately transferred to Qiagen Buffer AVL (Qiagen, Germantown, MD USA) for ribonucleic acid (RNA) extraction as per our institutional biosafety committee-approved protocol for working with field-collected samples which may contain a select agent. The remaining blood or blood in PBS was stored in serum separator tubes overnight before centrifugation at 1000 X g for 10 minutes. Serum was then transferred by pipette into a sterile microcentrifuge tube and frozen -80°C freezers or liquid nitrogen dry shippers the same night before long-term storage at -45°C.

### Molecular surveillance

Sample inactivations were performed with Qiagen Buffer AVL (Qiagen, Germantown, MD USA) and Ethanol as described in Haddock *et al*. 2016 [15] in a Class III biosafety cabinet located in the Laboratoire National de Santé Publique, Brazzaville, ROC. Oral/nasal, urogenital, and rectal swab samples were pooled for each individual bat sampled, 50 uL each with 140 uL then transferred for the RNA extraction, and extracted using the QIAamp Viral RNA extraction kit (Qiagen, Germantown, MD USA). Whole blood was extracted using the QIAamp Viral RNA extraction kit with an additional wash step (Qiagen, Germantown, MD USA). RNA was extracted from the liver, lung, and spleen using the RNeasy extraction kit (Qiagen, Germantown, MD USA) from 23 bats that were lethally sampled including 9 *Chaerephon pumilus* and 4 *Mops condylurus* bats from the Mossaka field site, and 5 *Epomops franqueti*, 2 *Hypsignathus monstrosus*, and 3 *Micropteropus pusillus* bats from the Liranga field site. A multiplexed, quantitative real-time polymerase chain reaction (qRT-PCR) was used to detect presence of EBOV and Bombali virus nucleic acids, modified from de Wit *et al*. 2016[16] to include primers and probes (Integrated DNA Technologies, Inc. Coralville, Iowa, USA) targeting the L gene for both Ebola virus (5′-CAGCCAGCAATTTCTTCCAT-3, 5′-TTTCGGTTGCTGTTTCTGTG-3′, FAM-ATCATTGGC/ZEN/RTACTGGAGGAGCAG-BHQ, FAM-TCATTGGCG/ZEN/TACTGGAGGAGCAGG-BHQ), Bombali virus (5’-TCTCGACGAAGGTCATTAGCGA-3’, 5’-TTGCTCTGGTACTCGCTTGGT-3’, FAM-TGCTGGGATGCTGTCTTTGAGCCT-BHQ) and bat HPRT (5’-AGATGGTCAAGGTCGCAAG-3’, 5’-CCTGAAGTATTCATTATAGTCAAGGG-3’, HEX-ACTTTGTTGGATTTGAAATTCCAGACAAGTTTG-BHQ) as an internal control. Briefly, 5 μL of extracted RNA was added per reaction with 20 μL of LightCycler 480 RNA Master Hydrolysis Probes reaction kit master mix (Roche, Indianapolis, IN; Product #04991885001). Thermal cycling included an initial reverse transcription step at 63°C for 3 minutes, an initial denaturation step at 95°C for 30 seconds, followed by 40 cycles of 95°C for 10 seconds, 60°C for 30 seconds, and 72°C for 6 seconds completed on a Magnetic Induction qPCR Cycler (MIC, Bio Molecular Systems, El Cajon, CA USA). A filovirus family-level reverse transcription-polymerase chain reaction (RT-PCR) was used to detect presence of filovirus nucleic acids as described in Schulz *et al*. 2020[17]. Full or partial *cytochrome b* was sequenced from 117 of 196 bats (accession numbers MZ265505-MZ265622) sampled to confirm morphological identification from the field. Species were confirmed based on greater than 98% sequence identity across 450 bp or 1,000 bp length sequences depending on whether longer amplicons could be recovered from the sample. Full or partial *cytochrome b* sequence was recovered for each bat using 3 μL of cDNA template, primers (Ducroz *et al*. 2001[18], long amplicons: 5’-ACCAATGACATGAAAAATCATCGTT-3’, 5’-TCTCCATTTCTGGTTTTACAAGAC-3’; or short amplicons targeting Molossinae bats: 5’-TACCATGAGGACAAATATC-3’, 5’-CAACTAGCAGTCAAAATAGA-3’, 5’-TAGATAGGACTAGGGCTAGT-3’) at 0.2 μM each in a 25 μL reaction with the CloneAmp HiFi PCR premix (Takara Bio USA, Inc, Mountain View, CA) and amplified with 35 cycles of 3-step PCR at 98°C for 10 seconds, 52°C for 5 seconds, and 72°C for 5 seconds.

### Serological surveillance

A commercial human anti-ZEBOV glycoprotein (GP) enzyme linked immunosorbent assay (ELISA) (Alpha Diagnostics, San Antonio, TX USA) was modified as follows; 100 μL bat serum diluted to 1:100 in PBS was added in duplicate to each well of the human anti-ZEBOV ELISA GP Immunoglobulin G (IgG) kit (#AE-320620-5) and incubated at room temperature for 1 hour, rinsed four times with the kit wash buffer before adding horseradish peroxidase-conjugated bethyl goat anti-bat IgG antibody at a 1:50,000 dilution and incubating for 30 minutes in an air-conditioned room at room temperature. The antibody was then washed four times with the kit wash buffer and trimethoprim substrate was added and allowed to incubate at room temperature for 15 min before 100 μL of stop solution was added and the optical density was measured at 450 nm on a BioRad iMark microplate reader. Threshold for seropositivity was determined as 3 times the standard deviation plus the mean OD of naive serum from captive bred *Rousettus aegyptiacus* bats.

## Results

A total of 196 bats were sampled; 165 bats from the Molosinnae subfamily sampled in Mossaka, ROC, and 30 fruit bats and a single bat from the Molosinnae subfamily was sampled in Liranga, ROC. No EBOV or filovirus genomic material was detected in any samples by targeted qRT-PCR or by pan-filovirus RT-PCR including lung, liver, and spleen samples from 23 lethally sampled bats, 172 blood samples, and pooled oral/nasal, urogenital, and rectal swabs from 185 individuals (S1 Table). We recovered serum samples from 172 of 196 bats. Serum samples were screened for reactivity against the EBOV glycoprotein; 8.1% of all bats sampled were seropositive with a cut-off of the mean OD plus 3 times the standard deviation of the naive controls (Table 1, S2 Table). We found higher seroprevalence among fruit bats in Liranga relative to the insectivorous bats sampled in Mossaka; however, fewer bats were sampled in Liranga (N = 28 and N = 144, respectively, Table 1). None of the *Micropteropus pusillus* bats were seropositive (N = 12), while both juvenile *Hypsignathus monstrosus* bats were seropositive and two of twelve adult *Epomops franqueti* bats were seropositive (Table 1). As we have fewer than 15 individuals sampled per species at the Liranga site, we did not perform bivariate analyses on this dataset. *Cytochrome b* sequence was not recovered for 49 of 145 Molossinae bats sampled, hindering species-level delineation for that group. Among the 145 Molossinae (*Mops* and *Chaerephon*) bats sampled in total, 54 were juvenile bats. While a lower proportion of juvenile Molossinae bats were seropositive relative to the adults (1 of 54 juvenile bats and 9 of 91 adult bats sampled, Table 1) the difference was not statistically significant by Chi-square Test and Fisher’s test executed in Prism version 9.1.1.

**Table 1:** Serological status (IgG) against EBOV glycoprotein in bats sampled in ROC near the Bikoro, DRC, Ebola virus disease outbreak in 2018.

Location	Species	Age	Seropositivity	Total
Mossaka (N = 144)	*Mops condylurus*	Adult	6.1% (2/33)	5.7% (2/35)
		Juvenile	0 (0/2)	
	*Chaerephon pumilus*	Adult	9.1% (2/22)	3.3% (2/61)
		Juvenile	0 (0/39)	
	Molossinae	Adult	13.9% (5/36)	12.5% (6/48)
		Juvenile	8.3% (1/12)	
Liranga (N = 28)	*Epomops franqueti*	Adult	15.4% (2/12)	15.4% (2/12)
		Juvenile	-	
	*Micropteropus pusillus*	Adult	0 (0/8)	0 (0/12)
		Juvenile	0 (0/4)	
	Molossinae	Adult	-	0 (0/1)
		Juvenile	0 (0/1)	
	*Hypsignathus monstrosus*	Adult	0 (0/1)	66.7% (2/3)
		Juvenile	100% (2/2)	
	Total	Adult	9.8% (11/112)	8.1% (14/172)
		Juvenile	5% (3/60)	

## Discussion

More than forty years have passed since the characterization of EBOV, including decades of ecological surveillance efforts, and still the wildlife reservoir remains elusive. Several surveillance efforts have reported on Ebolavirus seroprevalence in bats in Africa [19,20,21,22,23]. Using a multiplexed Luminex assay, LaCroix *et al*. (2021) reported a seroprevalence of 0.3–2.8% against at least one Ebolavirus antigen in the Bikoro region for bats sampled contemporaneously with this study, and none of the insectivorous bats were seropositive [23]. The two studies are complementary in sampling efforts with La Croix *et al*. (2021) having broadly sampled from frugivorous bats in the Équateur Province (>95% of samples) during the Bikoro EVD outbreak [23], while we sampled extensively from synanthropic insectivorous bats, *M*. *condylurus* and *C*. *pumilus*, which were found roosting in the wall cavities of human dwellings throughout Mossaka (S1 Fig, S1 Video). *M*. *chaerephon* and *C*. *pumilus* are widely distributed throughout the region and are commonly associated with human dwellings [24]. We observed the bats in the wall cavities of buildings including homes and the local hospital, suggesting opportunity for indirect contact with these bats at the human-animal interface.

Serological assays to detect Ebolavirus exposure history in bats are complicated by cross-reactivity [25,26,27], a lack of virus neutralizing antibodies generated in bats against filoviruses [28,29], and a short-lived detectable antibody response in bats following viral infection [29]. Gryseels and Mbala-Kingebeni *et al*. (2020) conducted extensive surveillance following the Likati EVD outbreak in DRC and reported no seropositive samples among rodents or bats using a multiplexed Luminex assay to broadly detect exposure to filovirus antigens [30]. Though our anti-EBOV IgG ELISA is likely to be less conservative and prone to cross-reactivity with exposure to related viral antigens (e.g. Bombali virus glycoprotein) than the multiplexed Luminex assays, our results are consistent with those of the Lacroix *et al*. (2021) study which likewise reported detection of seropositive bats sampled contemporaneously near the Bikoro 2018 EVD outbreak [23].

Responding rapidly to EVD outbreak situations in remote regions is a challenging endeavor and it is often months following the index case of an outbreak before an investigation of the initial spillover can take place. Despite rapid deployment of two independent field teams to conduct surveillance near the location of an EBOV spillover event, neither team recovered evidence of active infection in bats though both teams were able to detect antibodies against Ebolaviruses in bats in the region. There remain serious obstacles to uncovering the reservoir of Ebolaviruses in central Africa including 1) sampling deeply among relevant taxa in one of the most biodiverse regions on earth and 2) sampling during periods of viral shedding in wildlife populations which may be short-lived or associated with bat birthing pulses [3,31]. With an incubation period of 2–21 days after exposure and before symptom onset in humans [32], reactive sampling following declaration of an EVD outbreak may be asynchronous with a pulse of viral shedding in the wildlife reservoir/s.

In recent years, ecological data have been successfully integrated into trait-based statistical models to predict reservoir hosts for pathogens and optimize sampling strategies [33,34,35], though seasonality and timing of shedding events in wildlife remain less studied. Longitudinal surveys in wildlife populations including ecological context, seasonal variation, and improved understanding of regional human-domestic animal-wildlife interfaces are needed to refine sampling strategies to identify wildlife reservoirs of Ebolaviruses in the future. We emphasize the value of investment into in-country infrastructure and training for conducting surveillance activities and promoting early detection of spillover events as a critical component in both identifying periods and activities where spillover risk is highest and in mitigating outbreaks early following a spillover event [36,37].

## Supporting information

S1 Table Bat swab, blood, and tissue samples tested by qRT-PCR and Pan-filovirus RT-PCR. (TIFF)

S2 Table ELISA data for the seropositive samples and plate-specific serum controls collected from naïve Rousettus bats. (TIFF)

S1 Fig An opening (approximately 15 cm in length and 15 cm in height with wires covering part of the opening) in the wall of a hospital electrical room in Mossaka, Republic of the Congo, where insectivorous bats were found roosting. (TIFF)

S1 Video Bats exiting a roost in the walls of a structure in Mossaka, Republic of the Congo. (MOV)
