# Supplementary figures and images for: Zaire ebolavirus surveillance near the Bikoro region of the Democratic Republic of the Congo during the 2018 outbreak reveals presence of seropositive bats
Source: PLoS Negl Trop Dis. 2022 Jun 22;16(6):e0010504. doi: 10.1371/journal.pntd.0010504 (PMC9255767; doi:10.1371/journal.pntd.0010504)

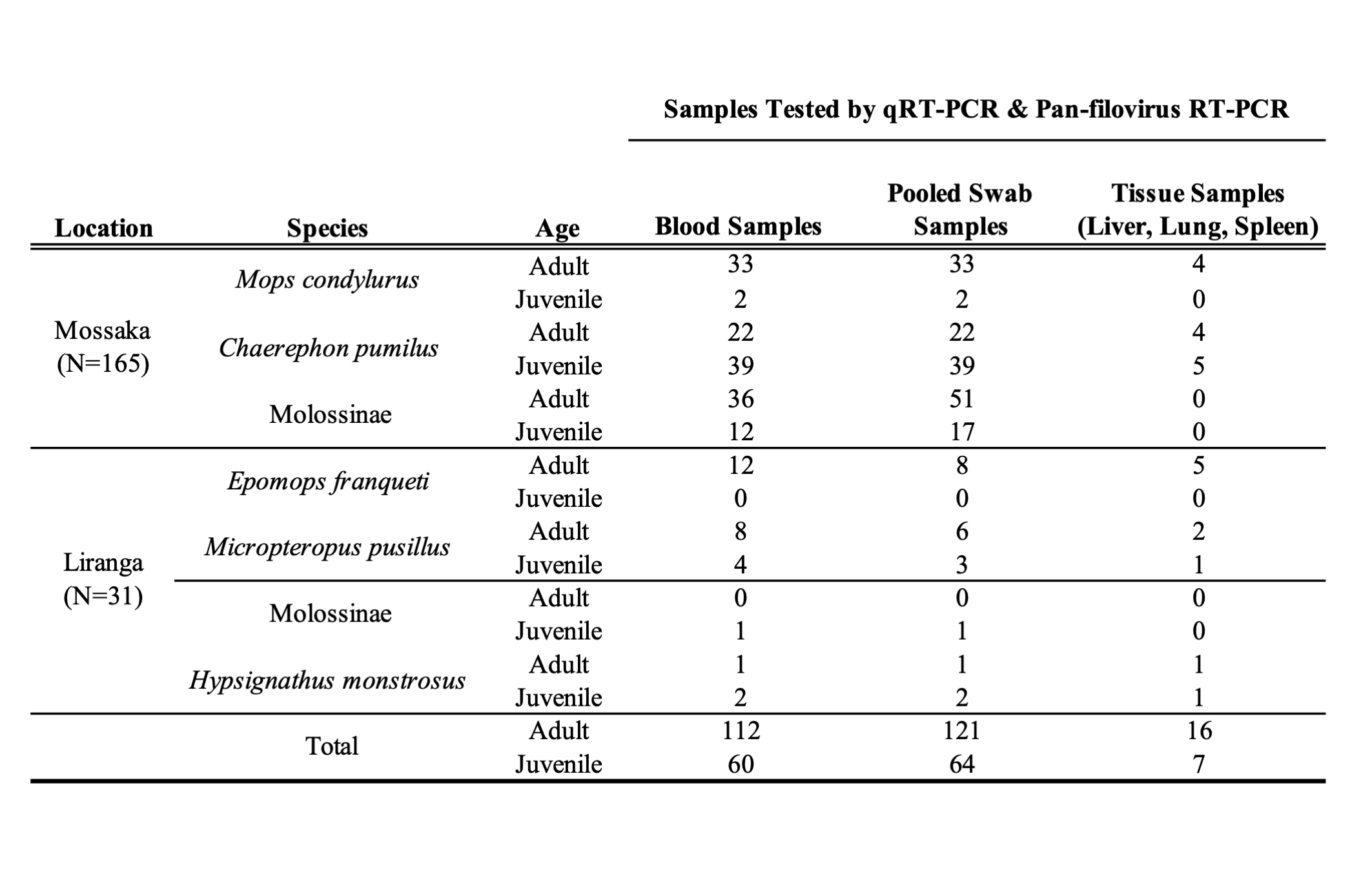

Supplement: S1 Table — (TIFF) [file pntd.0010504.s001.tiff]

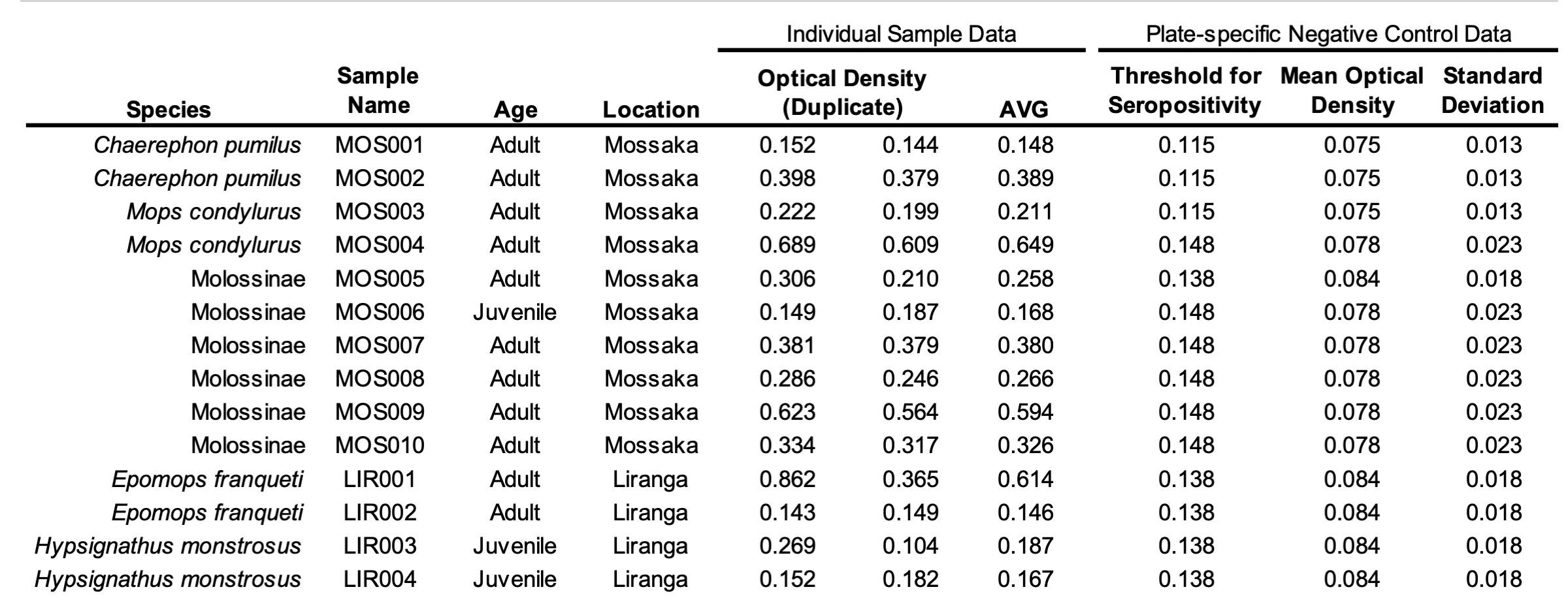

Supplement: S2 Table — (TIFF) [file pntd.0010504.s002.tiff]

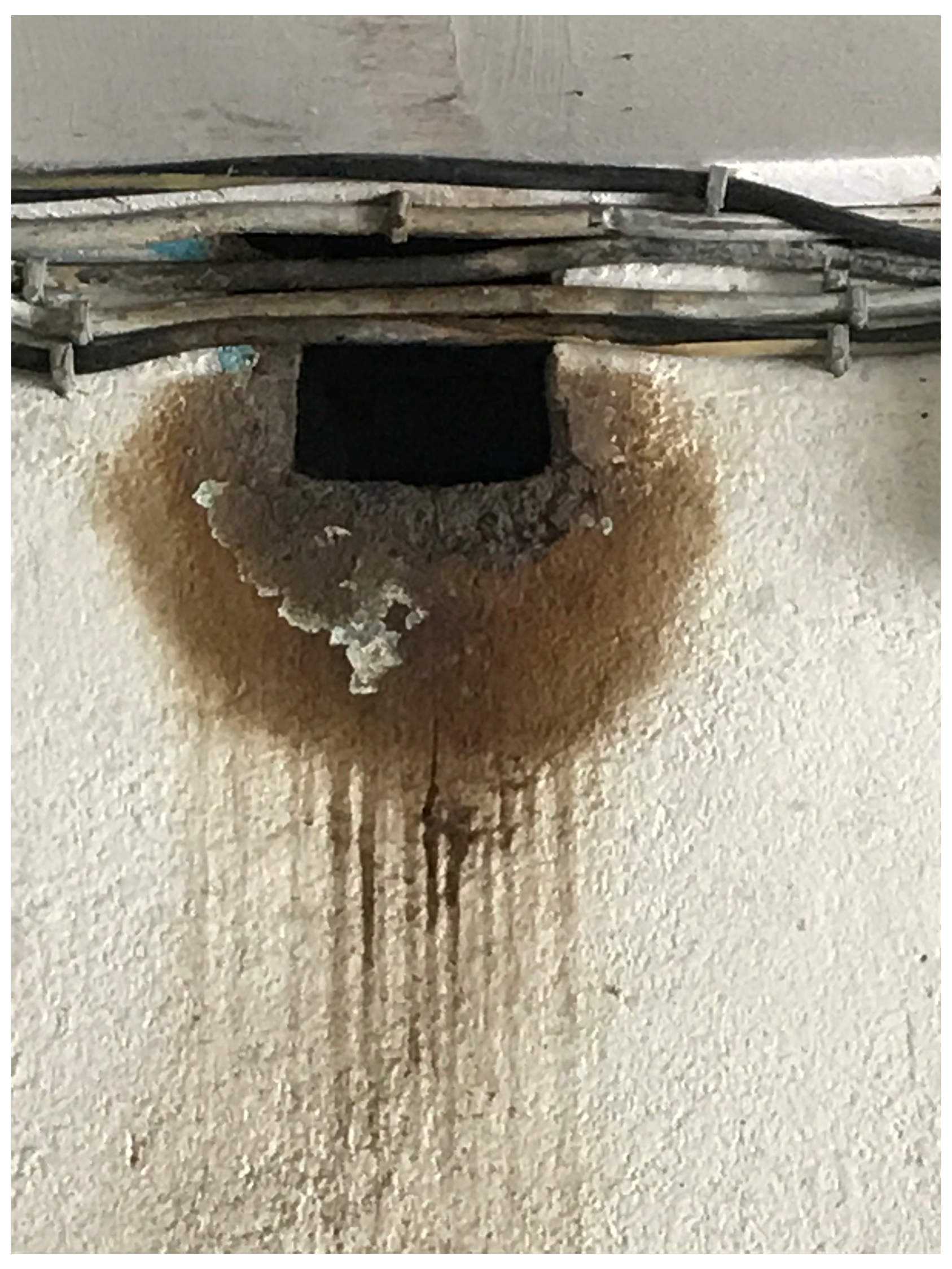

Supplement: S1 Fig — (TIFF) [file pntd.0010504.s003.tiff]
